# High-Resolution Multiscale Imaging Enabled by Hybrid Open-Top Light-Sheet Microscopy

**DOI:** 10.34133/2022/9761314

**Published:** 2022-08-13

**Authors:** Hong Ye, Guohua Shi

**Affiliations:** ^1^Jiangsu Key Laboratory of Medical Optics, Suzhou Institute of Biomedical Engineering and Technology, Chinese Academy of Science, Suzhou, China; ^2^School of Biomedical Engineering (Suzhou), Division of Life Sciences and Medicine, University of Science and Technology of China, Hefei, China

## 1. Introduction

Light-sheet fluorescence microscopy (LSFM), since it was first coined the term selective-plane-illumination microscopy (SPIM) by Huisken et al. [1], has been developed for nearly two decades, needless to mention a landmark development of digitally scanned light-sheet microscopy (DSLM) innovated by Keller et al. [2]. As a high-resolution optical sectioning imaging technique, LSFM has shown its superior ability for imaging transparent tissues at a fast volumetric imaging speed, thus widely used in developmental biology and neuroscience. With a strong desire to explore life sciences, the performance of LSFM has been continuously improved in many aspects, such as light-sheet engineering in pursuit of high resolution over a large field of view (FOV), sample installation ergonomics, advanced acquisition modalities, and efficient data processing [3]. In paper [4], Glaser et al. demonstrate a hybrid open-top light-sheet (OTLS) microscope that can rapidly screen centimeter-scale tissue volumes at several-micrometer resolution along with the ability to quantify millimeter-scale structures within the regions of interest (ROI) at the sub-micrometer resolution, opening up possibilities for many biological imaging applications.

**Figure 1:**
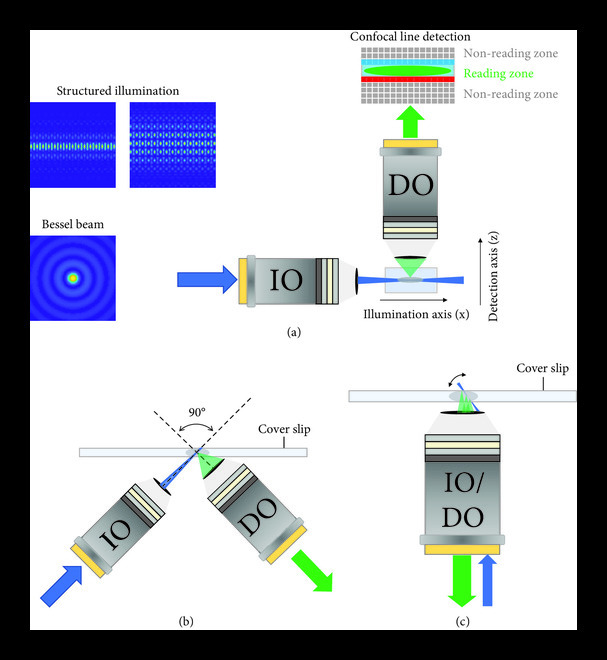
Sketches of (a) conventional LSFM system with an orthogonal illumination and detection configuration, along with a few approaches used to improve the LSFM performance during the development, *i.e*., Bessel beam and structured illumination in the illumination arm and confocal line detection in the detection terminal. (b) Open-top LSFM with an orthogonal configuration. (c) Single-objective LSFM with a nonorthogonal configuration. IO: illumination objective; DO: detection objective.

Conventional LSFM adopts an orthogonal dual-objective (ODO) configuration, which has the decoupled nature of the geometry in the excitation and collection optical paths, enabling the easy incorporation of many frontier photonics and imaging techniques into a working LSFM [5], as shown in Figure 1(a). While this geometry introduces lateral constraints to the specimen size and complicates the sample mounting, this issue can be ameliorated with an open-top configuration (see Figure 1(b)), but the resulting limited imaging depth, as well as the low tolerance of the index mismatch between the specimen and sample holder, is undesirable. Recently, the “single-objective” light-sheet microscopy (Figure 1(c)) features a nonorthogonal configuration (NODO), which is also compatible with a large diversity of modular sample holders, has gained popularity [6, 7]. Due to the lack of the objective lens of demanded but prohibitive specifications, neither of these systems can image millimeter-scale structures with sub-micrometer resolution. Glaser et al. report a system that combines a fast mesoscopic ODO path and a unique 45-degree microscopic NODO path, making use of two detection objectives and a shared illumination objective, to visualize biological structures with high-resolution multiscale imaging capability. Two representative imaging experiments have been spotlighted by Glaser et al. to express the impressive multiscale imaging ability of the present OTLS system. Utilizing this system, one can track the 100 nm thin axons of individual neurons in an intact mouse brain. Taking full advantage of the system, with the open-top configuration, Glaser et al. studied the unpredictable metastatic colonies from two cancer cell lines (MDA-MB-231 and OS-RC-2) through six intact mouse brains placed in a multiwell plate. Besides, an array of imaging experiments shown in the extended data and supplementary materials certify the hybrid OTLS system to be a powerful tool for versatile multiscale imaging applications.

The general approach to improving a light-sheet-based imaging system is using nondiffractive optical beams such as Bessel beam [8] or structured illumination [9, 10], to overcome scattering and extend the field of view. Since the present hybrid OTLS system leaves the excitation and detection arms uncoupled, the endless scope for customizing the illumination or detection arm is possible just like the conventional LSFM, while this in turn makes the system sophisticated and expensive to acquire. Despite many well-known imaging advantages, one reason that LSFM is popular among the biological community is that it is remarkably simple to be built, which allows easy access for biologists to verify their ideas experimentally. Though all LSFMs are ultimately based on either the SPIM or DSLM strategies [11], the enormous variants become bewildering for those looking to construct a light-sheet-based imaging system for a specific biological question. In the future, degrading the complexity of the system or forming a regular multifunctional system would be an important subject under discussion for all microscopists. Under this scope, Glaser et al. also discussed the theoretical possibility of using one single high-NA objective with a large FOV to achieve comparable performance.

The work of Glaser et al. is especially attractive given the growing demands of pursuing new biological insights, which often require higher spatiotemporal resolution in ever-larger and intact samples. Owing to the 2D sectioning nature of the light sheet, LSFM is an intrinsic fast imaging technique when the information from a single illuminated plane is simultaneously recorded on 2D array detectors such as CCD or sCMOS cameras. A fast volumetric imaging speed of up to 70 volumes/s has been reported [12]. Together with the deducted power intensity on the specimen and associated low photobleaching, the light-sheet-based imaging technology is ideal for revealing biological processes *in vivo*. A trite but inevitable issue that comes with it is data storage and processing. It is gratifying that the hybrid OTLS system efficiently decreases the data storage and imaging time via avoid taking tremendous data of the entire region but only the identified ROI. However, 180 GB data set of 24-minute recording as stated in [4] still pushes the storage capacity of even well-equipped laboratories. When it comes to acquiring images for an entire brain or animal, the data size soars up to petabytes. Data storage and transfer will impose a tremendous challenge on the researchers. A specially aimed facility may be a requisite for high-minded projects.

Another consideration that remains for LSFM imaging is the scatter of light in heterogeneous tissue which prevents researchers from achieving high-resolution 3D imaging of thick tissue and puts forward the demand for tissue clearing. All tissue clearing techniques, either solvent-based or aqueous-based, seek transparency through homogenizing the refractive index (RI) of a sample [13]. However, choosing an appropriate clearing technique for a specific scientific question requires careful evaluation of many parameters, such as sample size and the need for lipid staining and immunolabelling. Other concerns involve the usage of toxic solvents or specialized electrophoresis equipment. When centimeter-sized specimens are to be imaged, as in the case of Glaser’s paper, maintaining the constant axial resolution along the illumination axis should be considered first when selecting clearing techniques, then the RI matching between the specimen and objectives to ensure high resolution.

Future innovation aside, the report hybrid OTLS system has taken light-sheet-based imaging techniques to a new level, offering a novel way to obtain high-resolution multiscale optical sectioning detection at fast volumetric imaging speed with low photodamage and phototoxicity.
